# Neural Cell 3D Microtissue Formation Is Marked by Cytokines' Up-Regulation

**DOI:** 10.1371/journal.pone.0026821

**Published:** 2011-10-28

**Authors:** Yinzhi Lai, Amish Asthana, Ke Cheng, William S. Kisaalita

**Affiliations:** Cellular Bioengineering Laboratory, Department of Biological and Agricultural Engineering, Faculty of Engineering, Driftmier Engineering Center, University of Georgia, Athens, Georgia, United States of America; University of Pennsylvania, United States of America

## Abstract

Cells cultured in three dimensional (3D) scaffolds as opposed to traditional two-dimensional (2D) substrates have been considered more physiologically relevant based on their superior ability to emulate the in vivo environment. Combined with stem cell technology, 3D cell cultures can provide a promising alternative for use in cell-based assays or biosensors in non-clinical drug discovery studies. To advance 3D culture technology, a case has been made for identifying and validating three-dimensionality biomarkers. With this goal in mind, we conducted a transcriptomic expression comparison among neural progenitor cells cultured on 2D substrates, 3D porous polystyrene scaffolds, and as 3D neurospheres (in vivo surrogate). Up-regulation of cytokines as a group in 3D and neurospheres was observed. A group of 13 cytokines were commonly up-regulated in cells cultured in polystyrene scaffolds and neurospheres, suggesting potential for any or a combination from this list to serve as three-dimensionality biomarkers. These results are supportive of further cytokine identification and validation studies with cells from non-neural tissue.

## Introduction

Providing a 3D spatial microenvironment for cells to grow in, is the sole criterion that has traditionally been associated with three-dimensional cell culture. However, with recent advances in the field in the past decade, the meaning of 3D cell culture has been extended to providing the “total microenvironment” that supports the formation of microtissue that exhibits “complex” physiological relevance (CPR) or better emulation of the in vivo functionality in a manner not possible in 2D cultures [1]. Three main categories or microenvironment factors (MEFs) or “three-dimensions” from the literature include: 1) chemical or biochemical composition, 2) spatial (geometric 3D) and temporal dimensions, and 3) force and substrate physical properties [1]–[4]. However, there is still a lack of a quantifiable entity which can establish if the cellular response in a 3D culture is actually physiologically relevant and in vivo-like or just different from 2D. The identification and validation for this entity or a potential three-dimensionality biomarker is necessary due to three compelling reasons. First, apart from the concept of “three-dimensional matrix adhesion” originally proposed by Cukierman et al. [5] as a possible indication or “diagnosis” or marker for a culture state of three-dimensionality, the fields of tissue engineering and/or cell-based biosensors have not provided knowledge on the basis of which a consensus for three-dimensionality and the associated complex physiological relevance could be established. Because of this, claims of “physiologically more relevant” are readily made for cells cultured on any surface or scaffold that provides loosely defined 3D geometry, either at the nano- or micro- structure levels or their combinations, as long as the resulting cell phenotypes are different between the 2D and 3D geometries. Second, the concept of using combinatorial approaches to fabricate libraries of polymers or other material scaffolds [6], [7] for tissue engineering or cell-based drug discovery call for high throughput assay by which “hit materials” can be quickly identified for further development. Cell-material interaction outcome can potentially guide the development of such assays or biosensors [8]. An interaction with a material which yields cells that emulate in vivo conditions would be most desirable. Three-dimensionality biomarkers would provide the intellectual basis for material discovery platform development. Third, in order to lower the costs associated with 3D platforms and make them more accessible for high throughput screening (HTS) applications, simplification of the platform without giving up the physiologically relevant behavior of the cells is necessary, as discussed in detail by Lai et al. [4].

Taken together, the subfield or field of 3D culture needs ubiquitous validated biomarkers. As a first step, in search for three-dimensionality biomarkers, we initiated a cytokine expression comparative transcriptomic study with neural progenitor (NP) cells grown on 2D flat surfaces, 3D polymeric scaffolds and as neurospheres (NS). NS were used as the in vivo surrogate, since they have been shown to emulate many in vivo functions that have not been possible in 2D cultures [9], [10]. Cytokines are involved in many crucial cell functions like innate and adaptive inflammatory host defenses, cell growth, differentiation, cell death, angiogenesis, and development and repair processes [11]. Based on the structural homologies of their receptors they can be broadly classified into families like Colony Stimulating Factors, Interleukins, Interferons, TGF (transforming growth factor) family, TNF (tumor necrosis factor) superfamily, PDGF (platelet-derived growth factor) family and Chemokines [11]. Although cytokines have been extensively studied in the field of immunology and oncology, tissue or cell-based biosensor engineers have paid little attention to these small proteins that have potential to revolutionize the field. The evidence for their existence in 3D cultures is compelling but they have not yet been looked at as candidates for potential 3D biomarkers. However, they were an ideal family to explore for the search. The rationale behind their choice was based on the fact that, in a 3D microenvironment cells are surrounded by homotypic neighbors forming a loosely bound disorganized aggregate. When compared to in vivo, such a scenario exists only during avascular tumorogenesis or early stages of inflammatory wound healing and both these phenomenon are regulated by the same molecules – Cytokines [12]. So in vitro, the cells growing in 3D relate to any of those two models depending upon their type – malignant or primary, respectively, and therefore upregulation of their cytokine levels was physiologically relevant.

## Materials and Methods

### 2.1 Materials and reagents

Neural progenitor cells were obtained from Regenerative Bioscience Center at University of Georgia as well as Millipore (ENStem-A™, Billerica, MA). Polystyrene, chloroform, ammonium bicarbonate were obtained from Sigma (St. Louis, MO). Neural basal media, penicillin/streptomycin, L-glutamine, recombinant human leukemia inhibitory factor (hLIF), basic fibroblast growth factor (bFGF), B-27 supplement and phosphate buffered saline (PBS) were obtained from Gibco (Gaithersburg, MD). RNeasy mini kit was obtained from Qiagen (Valencia, CA). Human Genome U133 plus 2.0 microarray chips were obtained from Affymetrix (Santa Clara, CA).

### 2.2 Scaffold fabrication and neural progenitor cell culture

The scaffold fabrication process in Cheng et al. [13] was followed. Briefly, a viscous polymer solution was prepared by dissolving polystyrene (PS) in chloroform. Sieved ammonium bicarbonate particles in the range of 40–60 µm were added to the polymer solution and mixed thoroughly. These particles generated larger scaffold pore sizes from 60–100 µm, probably due to particle agglomeration. The paste mixture of polymer/salt/solvent was cast into the wells of a standard glass cell culture vessel. The optimal polymer/salt/solvent ratio of 1∶20∶5 (w/w/v) as determined in Cheng et al. [13] was used to generate the paste mixture. Casting was followed by chloroform evaporation, vacuum drying, and baking at 80°C overnight. Before use for cell culture, the scaffolds were sterilized by immersing in 70% alcohol overnight. PS scaffolds offer advantages of low cost, transparency for optical detection, and/or compatibility with existing instrumentation platforms in High Throughput Screening (HTS) applications.

Human neural stem cells or neural progenitors (NP) were maintained in neural basal media (Invitrogen, PA) supplemented with penicillin/streptomycin, L-glutamine, recombinant human leukemia inhibitory factor (hLIF), bFGF and B-27, at 37°C in a 5% CO_2_ humidified incubator. Medium was half changed every 48 hours. For differentiation, cells were allowed to grow until 90% confluent, after which they were exposed to differentiation medium which was half changed every 24 hours. The composition of differentiation medium was similar to the subculture medium described above with the exception of bFGF. For subculturing, 90% confluent cells were aspirated by pipetting and the subculture ratio was typically 1∶2 to 1∶3. Before cell seeding, both the 3D scaffolds and 2D substrates were coated with poly-ornithine and laminin to rule out any differences that may be caused by the polymer material itself. Neurospheres (NS) were formed by plating cells into non-coated dishes with shaking. Dishes without coating and shaking prevented NP cells from attaching to the surface and encourage them to attach to each other and form spheroid structures. The cell seeding density was 50,000 cells/cm^2^ for both 2D and 3D cultures.

### 2.3 Microarray gene expression analysis

Total RNA was isolated from all samples using Qiagen RNeasy Kits (Qiagen, Valencia, CA) according to the manufacturer's standard protocol. The quantity of mRNA isolated from each sample was determined using absorption at 260 and 280 nm. The purity of each sample was monitored using the A_260_/A_280_ ratio as well as housekeeping genes. A ratio of 1.8–2.1 was considered a “clean” sample and could be used in microarray experiments. Samples were sent to Affymetrix Core facility at Medical College of Georgia (MCG) for Human Whole Genome U133 plus 2.0 GeneChip Expression Analysis (Affymetrix, Santa Clara, CA). The expression data is publicly available on the GEO site as Series GSE13715.

The expression value of each gene was obtained by Expression Console (Affymetrix) with the RMA (Robust Multiple-array Average) algorithm. RMA is an algorithm widely applied to create expression values for Affymetrix data. The raw intensity values from Affymetrix genechips are background corrected, log2 transformed and then quartile normalized before a linear model was fit to obtain an expression measure for each probe set.

### 2.4 Statistical analysis

Linear discriminant analysis (LDA) with cross-validation was fulfilled by SAS DISCRIM procedure. Student t-test was used to compare the mean expression from two conditions (2D vs. 3D and 2D vs. NS) with significance level of 0.05. To determine whether cytokines as a group were significantly regulated by culture condition (i.e., 2D, 3D and NS), the permutation t-test method [14] was utilized.

## Results

The development of stem cell-based, HTS compatible, in vivo tissue-emulating biosensor platform requires a substrate or scaffold that is not only easy to fabricate but also provides stem cells with a 3D micro environment. For this purpose, 3D porous scaffolds were prepared from polystyrene (PS), a polymer material that has been widely applied in fabricating cell culture vessels. The physical and chemical properties of the scaffolds used in this study have been described by Cheng et al. [13]. Mouse superior cervical ganglion (SCG) and NP cells cultured in these scaffolds, and NP cells cultured as neurospheres (spheroids) have exhibited similar responses as freshly dissected SCG tissue in terms of voltage gated calcium channel and resting membrane potential, while 2D cultured cells exhibited significantly higher responses [13]. Based on Tuj staining (neuronal marker) the neural progenitor cells in scaffolds and neurospheres differentiated into nerve cells [15]. Additionally, spheroids have successfully emulated in vivo drug resistance [9], providing rationale for use of the neurospheres as an in vivo surrogate.

### 3.1 Quality of transcript expressions

Five experimental groups, with four biological replicates each, were chosen for this study. The five experimental groups were: 1) NP cells before differentiation induction cultured on 2D culture vessel (B2D), 2) NP cells before differentiation in 3D scaffolds (B3D), 3) one week culture of NP cells after differentiation induction on 2D surfaces (A2D), 4) one week culture of NP cells after differentiation in 3D scaffolds (A3D), and 5) neurospheres (NS). In order to minimize the cell passage and other environmental effects on cells, the samples were generated in 2D–3D paired manner, which means at least one 2D and one 3D sample were generated at the same time and from the same batch of cells. Intensity results of the transcripts were examined and normalized to exclude background signals and reported in arbitrary units using RMA (Robust Multiple-array Average) algorithm. RMA is an algorithm widely applied to create expression values for Affymetrix genechip data. The raw intensity values from Affymetrix genechips were first background corrected, log2 transformed and then quartile normalized. The final expression measure was obtained by fitting a linear model for each probe set on each array.

**Figure 1:**
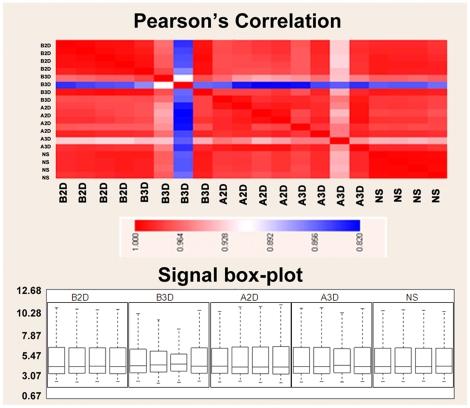
Microarray data quality. The quality of the microarray data was accessed by Pearson's correlation of the samples and the overall distribution of the mRNA expression. The upper panel shows the Pearson's correlation for each pair of the samples. B2D indicate the samples from “Before differentiation, 2D culture condition”, A2D “After differentiation, 2D culture”, B3D “Before differentiation, 3D culture”, A3D “After differentiation, 3D culture”, and NS “Neurospheres”. Pearson's correlation coefficient is a widely used similarity measure for gene expression data. It measures the similarity between two profiles by calculating the linear relationship of the distributions of the two corresponding random variables. The Pearson's correlation value is indicated the color bar color scale (values close to 1 indicate good correlation). The lower panel shows the box-plot of each sample. The box shows the range of the middle 50% with a line in the center for the median value. Additional lines indicate the overall range of the data.

Before we analyze the expression data, quality control tests were performed. No array results were accepted if the correlation coefficient (R^2^ value) between replicates was less than 0.95. Overall, samples exhibited good correlations with each other. Differentiation status and culture vessel difference didn't affect the overall gene expression pattern (Figure 1, top). We also examined the overall pattern of gene expression based on signal intensity box-plot (Figure 1, bottom). As is the case in box-plot data presentations, the lowest bar represents the observed sample minimum, the base of the box represents the lower quartile while the top represents the upper quartile, the bar inside the box represents the median, and the top bar represents the highest sample observation. The box-plot displays differences between populations without making any assumptions of the underlying statistical distribution. With the exception of a few samples, most of the five values represented in all the data sets (Figure 1, bottom), are in agreement. The third sample from B3D group (B3D_3) exhibited a very distinctive distribution of the expression value in which it had the lowest sample maximum and the smallest “distance” between upper quartile and lower quartile and had a very low correlation with other samples grown under similar conditions, as shown in Figure 1 (top). In order for array data to be accepted for analysis, the overall gene expression pattern has to be similar with no wide divergence between samples. Large variations indicate either hybridization errors or problems with the quality of the RNA used. For this reason B3D_3 was excluded from further analysis. Also, the third sample from A3D group had overall low expression values and a noticeable shift in intensity histogram (data not shown). It had a low correlation coefficient with sample grown under similar condition (Figure 1, top) and was also excluded from further analysis.

### 3.2 Cytokines' transcript and culture classification

The focus on cytokines in this study was based on previous proteomic and transcriptomic evidences from other 3D culture studies indicating that cytokines' expression levels are altered by culture conditions [16]–[19]. The list of cytokines analyzed was obtained from the Immunology Database and Analysis Portal (ImmPort) (https://www.immport.org/immportWeb/queryref/geneListSummary.do). There were a total of 681 probesets found in our microarray corresponding to the list of human cytokines. The permutation method was used to determine whether cytokines as a group was significantly regulated by culture conditions (i.e. 2D and 3D). Although we reduced the number of probesets to be analyzed from the genechip total of 54000 to 681, by focusing only on cytokines, the number of variables (transcript expression level in our case) still greatly exceeded the number of samples. Permutation testing is superior to standard tools for estimating statistical significance for multiple hypotheses testing due to its ability to empirically determine whether the observed class distinction could be obtained by chance. Briefly, the assay samples were re-iteratively reassigned randomly to the classification label (2D, 3D or NS). This process was done by randomly sampling function from R console's base package. After each reiteration, a list of *p* values were calculated measuring the significance of newly designated culture condition label for each probesets (2D vs. 3D or 2D vs. NS). After 1000 permutations, summary statistics (we used the median *p* value) with the permuted class distinction were generated and compared to those obtained in the experimental data [14], [20]. Results showed that cytokines as a group was significant when comparing 2D with 3D or NS (at a significance level of 0.05).

Next, we used linear discriminant analysis (LDA) to test if cytokine transcriptomic expressions can form a good criterion to separate 2D samples from 3D samples. LDA is a multivariate statistical technique commonly used to build a linear prescriptive or descriptive model which can characterize or classify each observation into two or more groups. LDA is also commonly used as a dimension reduction method for further classification analysis. Compared to cluster analysis such as Principle Component Analysis (PCA), LDA requires prior knowledge of the classes, which fitted our purpose of study.

The LDA results are presented in Table 1. As shown cytokines successfully classified 2D from 3D samples with acceptable low error counts. After-differentiation had a lower error rate in comparison to before-differentiation condition. This indicated that if 3D biomarker cytokines do exist, they may be culture time dependent. As pointed out by Lai et al. [4], under such circumstances biomarking with a profile as opposed to an end- point measurement would be the most practical and would increase the robustness of the biomarker. NS exhibited 0 error classification count, suggesting that it was more different from all other culture conditions.

**Table 1:** Classification of samples by linear discrimination analysis after cross-validation with 681cutokine probesets.

From	Number of samples classified to		Total	Error
	2D	3D		
2D	7	1	8	12.5%
3D	2	8	10	20%
Total	9	9	18	16.25%

From	Number of samples classified to						
	A2D	A3D	B2D	B3D	NS		
A2D	4	0	0	0	0	4	0
A3D	1	2	0	0	0	3	33.33%
B2D	0	0	3	1	0	4	25%
B3D	0	0	2	1	0	3	66.67%
NS	0	0	0	0	4	4	0
Total	5	2	5	2	4	18	25%

In Table 2 we list 16 cytokines selected by stepwise discriminant analysis with a cut off *p*-value of 0.01. With these 16 cytokines, the classification of 2D, 3D and NS is achieved with 0 error rate after cross-validation. Ten of these cytokines are commonly up-regulated in both 3D and NS culture conditions (however not statistically significant). Two are down-regulated both in 3D and NS. Although statistically these cytokines can discriminate 2D from 3D effectively, they are not as a group necessarily the best candidates for 3D biomarkers. This is because, the multivariate nature of the data makes the transcript expression levels correlated with each other, which means each of the variables (cytokine transcript expression levels in our case) can be represented by a group of other variables. Other cytokines may have the same or similar statistical power of these 16 cytokines to discriminate 2D from 3D, with better biological meanings. For this reason, cytokines significantly up-regulated in 3D and NS culture conditions were examined in detail.

**Table 2:** Most influential genes in LDA Classification by stepwise selection (p<0.01).

Symbol	Title	P value	Up regulated in:
GREM2	gremlin 2, cysteine knot superfamily, homolog (Xenopus laevis)	<.0001	
STC1	stanniocalcin 1	<.0001	3D and NS
GDF3	growth differentiation factor 3	0.0003	3D and NS
IFNA4	interferon, alpha 4	0.0008	3D and NS
NRG1	neuregulin 1	0.006	
INSL5	insulin-like 5	0.007	3D and NS
GHRL	Ghrelin/obestatin preprohormone	0.0012	3D and NS
LIF	leukemia inhibitory factor (cholinergic differentiation factor)	0.0059	NS
PPBPL2	pro-platelet basic protein-like 2	0.0017	3D and NS
FASLG	Fas ligand (TNF superfamily, member 6)	0.0041	3D and NS
CCL28	chemokine (C-C motif) ligand 28	<.0001	3D
FGF22	fibroblast growth factor 22	0.0016	NS
UCN3	urocortin 3 (stresscopin)	<.0001	3D and NS
CXCL2	Chemokine (C-X-C motif) ligand 2	0.0032	3D and NS
IFNA7	interferon, alpha 7	<.0001	3D and NS
EDN3	endothelin 3	0.005	3D

### 3.3 Cytokines up-regulated in 3D and NS

In Tables S1 and S2, we present cytokine significantly up-regulated (*p*≤0.05) in 3D and NS cultures. Forty and ninety-one probesets were up-regulated in 3D and NS culture conditions, respectively. The higher number in NS cultures is possibly due to inability to control the size of neurospheres. Many NS were observed to be larger than the 3D structure pore sizes (e.g., three times the maximum pore size of 100 µm) [13]. With large NS, the core of the cellular aggregate may experience hypoxia to the extent that genes not observed in 3D are up-regulated. Evidence in support of this comes from MIP-2 gene (inflammatory protein-2) induced by hypoxia [21] that was also found to be up-regulated in NS but not in 3D conditions in our study. Table 3 lists 13 genes (ANGTL7/CDT6, ARMET/MANF, BMP8B/OP2, CCL13/MCP-4, FGF5, GHRL, IL-11, IL-1B/IL-1F2, NOV/IBP-9, PDGFB, STC1, TGFA, and VEGF-A), whose transcripts were up-regulated in both 3D and NS culture conditions, and examples of their functions. We particularly focused on these genes in the following physiologic function discussion because they were less likely to be up-regulated because of conditions like hypoxia that may be present in NS but absent in 3D conditions.

**Table 3:** Cytokines up-regulated in both 3D and neurospheres with role examples in cells of nerve tissue origin.

Title	Symbol	Tumorogenesis	Inflammation	Development
angiopoietin-like 7/cornea-derived transcript 6	ANGPTL7/CDT6	Reduces tumor growth & acts as a negative regulator of angiogenesis in corneal cells [29].		Maintenance of corneal avascularity [29].
arginine-rich, mutated in early stage tumors/mesencephalic astrocyte-derived neurotrophic factor	ARMET/MANF	Inhibits tumor cell proliferation under hypoxia induced ER stress & protects tumor cells from ER stress-induced death [30].	Protects neurons from ER stress. Promotes neuron proliferation & prevents apoptosis during neuro- degeneration [31]	Expressed in developing nigro-striatal system at P1 & P10, suggesting a role in development of midbrain dopaminergic neurons [32].
Bone morphogenetic protein 8b (osteogenic protein 2)	BMP8B			
chemokine (C-C motif) ligand 13	CCL13			
fibroblast growth factor 5	FGF5	Oncogenic activities in astrocytic tumours by promoting growth, survival and migration & supporting neoangiogenesis [33].		Regulates neuron differentiation, survival [34], as well as astroglial properties [35].
Ghrelin/obestatin preprohormone	GHRL	Regulates tumor proliferation [36].		
interleukin 11	IL11			Neuropoietic effect on neurons [37]. Astrocyte [38] & neuronal differentiation [39].
interleukin 1, beta	IL1B	Expressed by glial cells around a tumor that are involved in immune reactions against the tumor & the damage caused by it [40].	Pro-inflammatory causes neural damage after CNS injury by inducing nitric oxide, free radicals & neurotoxins. Induces astrocytes to produce GFs that affect survival & proliferation of oligodendrocytes [41].	Inducer of remyelination [42].
nephroblastoma overexpressed gene	NOV	Associated with tumorogenesis, tumor differentiation, metastasis [43] & angiogenesis [44].	Regulates angiogenesis and fibroblast functions during wound healing [45].	Expressed in early stages (E3) neuroepithelium and later stage (E3–E7) neural tube [46]. Detected highly in human neuronal cells and axons [47]. Embryonic vascular development [44].
platelet-derived growth factor beta polypeptide(simian sarcoma viral (v-sis) oncogene homolog)	PDGFB	Induces the formation & progression of gliomas in neural progenitor cells. Required to overcome cell-cell contact inhibition and confers in vivo infiltrating potential to tumor cells [48].	Released by astrocytes and neurons after injury. Important for neuroprotection and repair in connection with neural disease and injury [49].	Neuronal development and diffentiation of undifferentiated NE cells directly to neurons [50].Increases survival and neurite outgrowth of fetal striatal neurons [51].
stanniocalcin 1	STC1	Marker of human cancer. Regulates tumor size, proliferation & micrometastases [52].	Protects neurons from oxidative & hypoxic stress [53].	Regulates terminal differentiation of neural cells [54].
transforming growth factor, alpha	TGFA	Mitogenic for glioma cell lines. Participates in angiogenesis of glioma by inducing expression of VEGF [55].		Regulates neural progenitors proliferation/cell fate choice, neuronal survival/differentiation, astrocytic reactivity & has neurotrophic effects on neurons [56].
vascular endothelial growth factor A	VEGFA	Induces angiogenesis, promotes cell migration & invasion potential of glioma cells 57,58.	Role in blood-brain barrier (BBB) breakdown and angiogenesis after brain injury [59]. Astrocytes in the perilesional area express VEGF-A early after injury [60].	Shows angiogenic, blood–brain barrier permeabilizing, neurotrophic, gliotrophic, and anti-apoptotic actions. [61].

## Discussion

Consistent with our results, up-regulation of cytokines in 3D cultures compared to 2D has been reported by several transcriptomic studies using cells from the four main tissue types (nerve, muscle, connective, and epithelial) cultured in a wide variety of platforms. For example, Klapperich and Bertozzi [19] showed that seven cytokines (IL-8, CXCL1, CXCL2, CXCL3, CXCL5, VEGF, LIF) were up-regulated in human fetal lung fibroblasts (IMR-90) cultured in a collagen–glycosaminoglycan (collagen/GAG) 3D mesh when compared to 2D surfaces. Also, up-regulation of six cytokines (CXCL1- 3, IL-8, MIP-3a, Angiopoetin like4) by a melanoma cell line (NA8) cultured on poly-2-hydroxyethyl methacrylate (polyHEMA) plates was reported by Ghosh at al. [18]. Transcriptomic findings such as those in the above examples have been further substantiated by studies at the protein level. For example, Enzerink et al. [16] have reported induction of chemokine (CCL2-5, CXCL1-3, CXCL8) secretion due to clustering of cells in five different fibroblast cell lines cultured in agarose. Also, Fischbach et al. [17] cultured tumor cells (oral squamous cell carsinoma) in a 2D and 3D RGD-alginate system and reported a dramatic enhancement of IL-8 levels in 3D. Another study by the same group showed that when the same cells were grown in Matrigel (lrBM) there was up-regulation of cytokines when compared to 2D. This observation is of particular importance as cells grown on Matrigel have already been shown to produce an outcome similar to in vivo, like the formation of mammary gland acinus and milk-like secretion into lumen [22] proving that Matrigel can provide all the relevant microenvironmental factors. This suggests that the up-regulation of cytokines in 3D compared to 2D is not a random differential response but is pertinent as a similar response is elicited when a proven physiologically relevant microenvironmental platform is provided.

We have used the themes of tumorogenesis, inflammation and development as shown in Table 3 to closely examine the13 up-regulated transcripts in both 3D and NS culture conditions; cells in a 3D culture in vitro relate to in vivo phenomenon like avascular tumor progression, early stages of inflammatory wound healing or embryonic development depending upon their type- malignant, primary or stem [4]. As these conditions are regulated by autocrine and paracrine cytokine signaling in vivo, the up-regulation of cytokines in 3D culture is physiologically relevant.

Angiogenesis is recurrent function among the genes listed in Table 3, which is not surprising. As already mentioned, as a microtissue grows beyond a certain size, nutrient and oxygen depletion become limiting factors leading to the inhibition of cell proliferation and initiation of angiogenic signaling. Oxygen concentration in 3D tissues depends on the balance between oxygen delivery and consumption. In vivo, this balance is tightly regulated by evenly distributed capillary networks but in vitro homotypic 3D microtissues lack vasculature and therefore develop a hypoxic core as their size increases. This event leads to the cells producing chemical signals (cytokines) for angiogenesis and is quite similar to the response occurring in normal hypoxic tissues where balanced signaling cascades lead to vascular remodeling and angioadaptation until the tissue oxygen concentration is back within its normal range [23]. The up-regulation of VEGF-A and other genes with angiogenic function like ANGPTL7/CDT6, FGF5, NOV/IBP-9, and TGFA is pointing to a functional class of cytokines with great potential as three-dimensionality biomarkers.

The above genes may play other roles besides being factors involved with hypoxia induced angiogenesis. For example, VEGF-A has been shown to regulate neuronal development, survival, neurite growth and it also possesses gliotrophic properties. Also, it's up-regulation is not limited to neuronal cells; it has been shown to be up-regulated in 3D cultures of a variety of cell and tissue types including human fetal lung fibroblasts [19], oral squamous cell carcinoma, glioblastoma, breast cancer [17], neonatal rat cardiomyocytes and neonatal mouse cardiomyocytes [24]. This up-regulation in a non-cell type specific manner in 3D cultures of VEGF-A, and probably other members of the class, lends credibility to the notion of ubiquity of these potential biomarkers in different cells derived from different tissue types.

Another recurrent gene function theme in Table 3 is terminal differentiation, a process by which cells commit to being part of a particular tissue or organ and perform a particular function. Terminal differentiation is preceded by inhibition of proliferation and cell cycle arrest. This exerts endoplasmic reticulum stress on the cell, but anti-apoptotic factors like Bcl-2 protect the cell from apoptosis and commit it to differentiation. In 3D, much like in organs in vivo, there is a spatial constraint on the microtissue, exerted by the defined pore size of the scaffold, which prevents them from proliferating freely, maintaining them in a quiescent state. Evidence in support of this comes from the fact that cells grown in 3D have shown lower proliferation rates than their 2D counterparts and higher expression of cyclin dependent kinase inhibitors (CDI) p21 [25]. Also integrin mediated adhesion to the ECM leads to activation of Bcl-2 family of genes and makes the 3D cells more resistant to apoptosis [26]. Such conditions are favorable for the cell to undergo terminal differentiation and this can be clearly seen by up-regulation in a number of differentiation and survival factor in 3D and NS compared to 2D, like PDGFB and STC1. Neural progenitor cells cultured in 3D have been shown to differentiate and produce a heterogeneous population consisting of neurons, astrocytes and oligodendrocytes [27]. Such cultures essentially behave as a coculture where cells are in symbiotic relationship with each other and might produce factors that can modulate the functions of the other cell types. This has been confirmed by intermediate filament glial fibrillary acidic protein (GFAP), a marker for astrocytic differentiation that was up-regulated in 3D and NS compared to 2D cultures (data not shown). It is a well known fact that astrocytes play a trophic role in supporting neurons. Factors secreted by astrocytes generally belong to the FGF, TGF and EGF families that play an important role in early neurogenesis [28]. Members of these families act as potent mitogens for multipotential neural progenitors and have been implicated in the regulation of several aspects of neurogenesis. Therefore up-regulation of members of these cytokine families in 3D and NS in this study is not surprising. The identification and validation of a few cytokines as three-dimensionality biomarkers that are ubiquitous among cells from different tissue types needs to be done.

Overall, cytokines gene expression results in this study support the notion that 3D cultured cells in various formats are different from their 2D counterparts. Furthermore, up-regulated cytokines' transcripts, independent of culture format, have been identified; this group of 13 cytokines commonly up-regulated in cells cultured in polystyrene scaffolds and neurospheres are suggesting potential for any or a combination from this list to serve as three-dimensionality biomarkers. These results are supportive of further cytokine identification and in vitro/in vivo validation studies with cells from non-neural tissue.

## Supporting Information

Table S1 **Significantly up-regulated genes in 3D.**
(DOCX)

Table S2 **Significantly up-regulated genes in neurospheres.**
(DOCX)
