# Supplementary material for: Neural Cell 3D Microtissue Formation Is Marked by Cytokines' Up-Regulation
Source: PLoS One. 2011 Oct 28;6(10):e26821. doi: 10.1371/journal.pone.0026821 (PMC3203927; doi:10.1371/journal.pone.0026821)
Supplement: Table S2 — Significantly up-regulated genes in neurospheres. (DOCX) [file pone.0026821.s002.docx]

Table S2. Significantly up-regulated genes in neurospheres.

| Title | Symbol | *P*-value | Affymetrics Probeset ID |
| --- | --- | --- | --- |
| Adrenomedullin | ADM | 1E-06 | 202912_at |
| Adrenomedullin 2 | ADM2 | 0.016 | 220538_at |
| Angiopoietin-like 7 | ANGPTL7 | 0.054 | 206423_at |
| arginine-rich, mutated in early stage tumors | ARMET | 0.0002 | 202655_at |
| bone morphogenetic protein 5 | BMP5 | 3E-05 | 205431_s_at |
| Bone morphogenetic protein 8b (osteogenic protein 2) | BMP8B | 0.050 | 235275_at |
| chromosome 19 open reading frame 10 | C19orf10 | 0.0002 | 216483_s_at |
| chromosome 19 open reading frame 10 | C19orf10 | 0.001 | 221739_at |
| complement component 5 | C5 | 0.002 | 205500_at |
| chemokine (C-C motif) ligand 13 | CCL13 | 0.040 | 216714_at |
| chemokine (C-C motif) ligand 23 | CCL23 | 0.040 | 210548_at |
| CD320 molecule | CD320 | 0.037 | 218529_at |
| chemokine-like factor | CKLF | 0.0005 | 219161_s_at |
| chemokine-like factor | CKLF | 0.049 | 221058_s_at |
| C-type lectin domain family 11, member A | CLEC11A | 0.002 | 211709_s_at |
| CKLF-like MARVEL transmembrane domain containing 3 | CMTM3 | 0.018 | 1555705_a_at |
| CKLF-like MARVEL transmembrane domain containing 3 | CMTM3 | 0.004 | 224733_at |
| CKLF-like MARVEL transmembrane domain containing 6 | CMTM6 | 0.034 | 217947_at |
| CKLF-like MARVEL transmembrane domain containing 6 | CMTM6 | 0.009 | 223047_at |
| CKLF-like MARVEL transmembrane domain containing 7 | CMTM7 | 0.0003 | 226017_at |
| chemokine (C-X-C motif) ligand 10 | CXCL10 | 0.013 | 204533_at |
| Chemokine (C-X-C motif) ligand 2 | CXCL2 | 0.033 | 230101_at |
| defensin, beta 4 /// defensin, beta 4, pseudogene | DEFB4 /// DEFB4P | 0.020 | 207356_at |
| epidermal growth factor (beta-urogastrone) | EGF | 0.006 | 206254_at |
| family with sequence similarity 3, member C | FAM3C | 0.014 | 240062_at |
| fibroblast growth factor 11 | FGF11 | 0.0002 | 227271_at |
| Fibroblast growth factor 14 | FGF14 | 0.0006 | 230231_at |
| Fibroblast growth factor 14 | FGF14 | 0.024 | 230288_at |
| fibroblast growth factor 20 | FGF20 | 0.039 | 220394_at |
| fibroblast growth factor 22 | FGF22 | 0.048 | 221315_s_at |
| fibroblast growth factor 5 | FGF5 | 0.022 | 208378_x_at |
| fibroblast growth factor 6 | FGF6 | 0.040 | 208417_at |
| galanin | GAL | 0.007 | 207466_at |
| galanin | GAL | 0.005 | 214240_at |
| growth differentiation factor 10 | GDF10 | 0.003 | 206159_at |
| growth differentiation factor 15 | GDF15 | 0.003 | 221577_x_at |
| glial cell derived neurotrophic factor | GDNF | 0.025 | 230090_at |
| Ghrelin/obestatin preprohormone | GHRL | 0.019 | 237647_at |
| glia maturation factor, gamma | GMFG | 0.004 | 204220_at |
| interferon, alpha 14 | IFNA14 | 0.021 | 208182_x_at |
| interferon, alpha 16 | IFNA16 | 0.025 | 208448_x_at |
| interferon, alpha 17 | IFNA17 | 0.018 | 211405_x_at |
| interferon, alpha 21 | IFNA21 | 0.012 | 211145_x_at |
| Interleukin 11 | IL11 | 0.034 | 206926_s_at |
| interleukin 19 | IL19 | 0.034 | 220745_at |
| interleukin 1, beta | IL1B | 0.048 | 39402_at |
| inhibin, beta E | INHBE | 0.005 | 210587_at |
| jagged 2 | JAG2 | 0.004 | 209784_s_at |
| jagged 2 | JAG2 | 6E-05 | 32137_at |
| midkine (neurite growth-promoting factor 2) | MDK | 0.0005 | 209035_at |
| macrophage migration inhibitory factor (glycosylation-inhibiting factor) | MIF | 0.0002 | 217871_s_at |
| pre-B-cell colony enhancing factor 1 | PBEF1 | 0.0001 | 1555167_s_at |
| pre-B-cell colony enhancing factor 1 | PBEF1 | 2E-05 | 217738_at |
| pre-B-cell colony enhancing factor 1 | PBEF1 | 4E-06 | 217739_s_at |
| nephroblastoma overexpressed gene | NOV | 0.027 | 214321_at |
| nephroblastoma overexpressed gene | NOV | 0.0008 | 204501_at |
| neuropeptide Y | NPY | 0.017 | 206001_at |
| nudix (nucleoside diphosphate linked moiety X)-type motif 6 | NUDT6 | 0.004 | 220183_s_at |
| nudix (nucleoside diphosphate linked moiety X)-type motif 6 | NUDT6 | 0.0008 | 230329_s_at |
| osteoglycin | OGN | 0.038 | 218730_s_at |
| platelet-derived growth factor beta polypeptide (simian sarcoma viral (v-sis) oncogene homolog) | PDGFB | 0.036 | 216061_x_at |
| platelet-derived growth factor beta polypeptide (simian sarcoma viral (v-sis) oncogene homolog) | PDGFB | 0.043 | 204200_s_at |
| platelet derived growth factor D | PDGFD | 1.98E-07 | 219304_s_at |
| platelet derived growth factor D | PDGFD | 0.002 | 222860_s_at |
| prokineticin 1 | PROK1 | 0.022 | 229124_at |
| pleiotrophin (heparin binding growth factor 8, neurite growth-promoting factor 1) | PTN | 0.010 | 208408_at |
| pleiotrophin (heparin binding growth factor 8, neurite growth-promoting factor 1) | PTN | 0.001 | 209465_x_at |
| pleiotrophin (heparin binding growth factor 8, neurite growth-promoting factor 1) | PTN | 0.008 | 209466_x_at |
| pleiotrophin (heparin binding growth factor 8, neurite growth-promoting factor 1) | PTN | 0.018 | 211737_x_at |
| relaxin 2 | RLN2 | 0.008 | 214519_s_at |
| small inducible cytokine subfamily E, member 1 (endothelial monocyte-activating) | SCYE1 | 0.005 | 202541_at |
| small inducible cytokine subfamily E, member 1 (endothelial monocyte-activating) | SCYE1 | 5.11E-07 | 202542_s_at |
| small inducible cytokine subfamily E, member 1 (endothelial monocyte-activating) | SCYE1 | 0.0007 | 227605_at |
| sema domain, immunoglobulin domain (Ig), short basic domain, secreted, (semaphorin) 3C | SEMA3C | 0.005 | 203788_s_at |
| sema domain, immunoglobulin domain (Ig), short basic domain, secreted, (semaphorin) 3C | SEMA3C | 0.0005 | 203789_s_at |
| stanniocalcin 1 | STC1 | 1E-05 | 204597_x_at |
| stanniocalcin 1 | STC1 | 0.0256 | 204595_s_at |
| stanniocalcin 1 | STC1 | 1E-07 | 204596_s_at |
| Stanniocalcin 1 | STC1 | 7E-05 | 230746_s_at |
| stanniocalcin 2 | STC2 | 0.0006 | 203438_at |
| stanniocalcin 2 | STC2 | 0.001 | 203439_s_at |
| transforming growth factor, alpha | TGFA | 0.005 | 205016_at |
| thrombopoietin (myeloproliferative leukemia virus oncogene ligand, megakaryocyte growth and development factor) | THPO | 0.040 | 211831_s_at |
| tumor necrosis factor receptor superfamily, member 11b (osteoprotegerin) | TNFRSF11B | 0.019 | 204933_s_at |
| tumor necrosis factor (ligand) superfamily, member 9 | TNFSF9 | 0.007 | 206907_at |
| taxilin alpha | TXLNA | 0.042 | 1561358_at |
| urotensin 2 domain containing | UTS2D | 0.047 | 243175_at |
| vascular endothelial growth factor A | VEGFA | 3E-07 | 210513_s_at |
| vascular endothelial growth factor A | VEGFA | 0.002 | 210512_s_at |
| vascular endothelial growth factor A | VEGFA | 0.011 | 211527_x_at |
| vascular endothelial growth factor A | VEGFA | 0.0006 | 212171_x_at |
| VGF nerve growth factor inducible | VGF | 0.003 | 205586_x_at |
